# Electrical properties of single CdTe nanowires

**DOI:** 10.3762/bjnano.6.45

**Published:** 2015-02-12

**Authors:** Elena Matei, Camelia Florica, Andreea Costas, María Eugenia Toimil-Molares, Ionut Enculescu

**Affiliations:** 1 National Institute for Materials Physics, Magurele, Ilfov, Romania, 77125; 2 GSI, Planckstraße 1, 64291 Darmstadt, Germany

**Keywords:** CdTe, electrodeposition, nanowires, transport properties

## Abstract

Ion track, nanoporous membranes were employed as templates for the preparation of CdTe nanowires. For this purpose, electrochemical deposition from a bath containing Cd and Te ions was employed. This process leads to high aspect ratio CdTe nanowires, which were harvested and placed on a substrate with lithographically patterned, interdigitated electrodes. Focused ion beam-induced metallization was used to produce individual nanowires with electrical contacts and electrical measurements were performed on these individual nanowires. The influence of a bottom gate was investigated and it was found that surface passivation leads to improved transport properties.

## Introduction

Nanowires, which are quasi one-dimensional structures, are considered an extremely important class of nanostructures, regarded as highly effective building blocks for future electronic devices [1–4]. In addition to their specific dimensions and high aspect ratio (which enable ultraminiaturization, due to the high surface-to-volume ratio), nanowires provide increased functionality for electronic devices which contain them [5–7].

There are numerous ways to fabricate nanowires, with methods ranging from simple and straightforward wet chemistry approaches, to complex multistep approaches that were developed over the past two decades. The main goal in all experiments involving such nanostructure preparation is to control both the morphological properties and the structural and compositional characteristics, in order to control the functionality of the nanowires. It is also important that the fabrication method leads to reproducible results and is highly scalable, thus increasing the efficiency of the preparation step.

The template approach is a method which enables the fabrication of nanowires with excellent reproducibility and a narrow distribution of the geometrical characteristics [8–13]. The method typically makes use of a nanoporous membrane as a template along with a method for filling its pores. As templates, most used are polymer ion track membranes, anodic alumina and diblock copolymer templates, while the filling methods range from electrochemical or electroless deposition, to atomic layer deposition or molten metal injection.

The nanoporous polymer ion track membranes are obtained by polymer foil irradiation with swift heavy ions and further chemical etching of the ion tracks [13]. This method allows for control of pore density by taking into account that each ion leaves a single, cylindrical track and pore size throughout the etching process. These parameters are usually chosen in connection with the desired final nanowire size and quantity.

Electrochemical deposition is a well-established method of plating conductive substrates with a specific metal or alloy. During the last decades, semiconductor electrodeposition became more and more attractive, as it may represent a viable alternative to more expensive fabrication methods [14–16]. CdTe electroplating is an excellent example of semiconductor electrodeposition for both film and nanostructure fabrication [14]. By employing a bath containing both cadmium and telluride, their controlled reduction at the working electrode leads to the formation of a high quality, stoichiometric, compound semiconductor.

In this work, a template approach for fabricating CdTe nanowires by electrodeposition inside ion track polycarbonate nanoporous membranes was employed. It was recently proved that one can easily control the characteristic of the nanowires prepared in this way by controlling the electrodeposition overpotential [15]. However, the number of reports dealing with the electrical properties of individual CdTe nanowires are very few.

In the present report, in addition to basic characterization regarding morphology, structure and composition determination, the nanowires were connected with electrical contacts by means of a combination of lithography and focused ion beam-induced metallization (FIBIM). The electrical properties were determined for individual nanowires prepared under different conditions. Further, the effect of a bottom gate on the charge carriers transported through the nanowire channel was examined. It was also found that (similar to other cases of semiconductor nanowires) the surface passivation leads to an improvement in the electrical properties [16–17].

## Results and Discussion

Electrochemical deposition of CdTe is a process that has been studied over several decades, and is one of the first reports of an electrodeposited semiconductor. The mechanism leading to the formation of the stoichiometric compound was thermodynamically explained based on the free energy corresponding to the compound formation reaction. In the case of this particular compound, this free energy opens up the possibility to obtain the stoichiometric composition for a rather wide range of electrode potentials and the ability to tune the bath composition over a rather wide range. Practically, the electrodeposition of multicomponent materials (either alloys or compounds) is related to the differences in reduction potentials for each component. In order to reach the desired composition, the deposition conditions must be adjusted in order to reach the desired reaction rate for each component. The procedure typically involves a deposition bath containing a high ratio of the element that is reduced at more electronegative values to the element that is reduced at less electronegative values. For the electrodeposition of CdTe, a bath containing CdSO_4_ as a source of Cd ions and TeO_2_ as a source of Te ions was employed at a ratio of 100 (considering that Cd is reduced at a far more negative electrode potential than Te). Consequently, the electrochemical polarization curves show a plateau in current over a range covering approximately 300 mV.

Electrodeposition inside nanoporous membranes has several particularities, which are a consequence of the fact that the process takes place in a restricted geometry ([13] gives a detailed description of the process). In this regard, the diffusion of ions through the nanopores is different from typical diffusion in an open bath when plating on a typical two-dimensional electrode. As a consequence, the current versus time curve shows a strong current increase when the pore is completely filled and during switching from deposition inside the nanopore to deposition on the surface. This effect was used to determine the time necessary for complete filling whereby the process can be stopped earlier. The nanowires growing from caps on the surface (indicating complete pore filling) are more difficult to harvest and contact, and therefore, the deposition process should be stopped before cap formation.

The deposition was performed in membranes with 10^8^ and 10^9^ pores/cm^2^ and different pore diameters. In order to characterize the nanowires, the template membrane was dissolved by immersing the template containing the nanowires in chloroform and the process was repeated at least 5 times. The process was started with p.a.-grade chloroform and for the last two washing steps, semiconductor-grade chloroform was used. Thorough washing is important for precise electrical characterization since template remnants can influence the characteristics of the metal–semiconductor interface.

**Figure 1:**
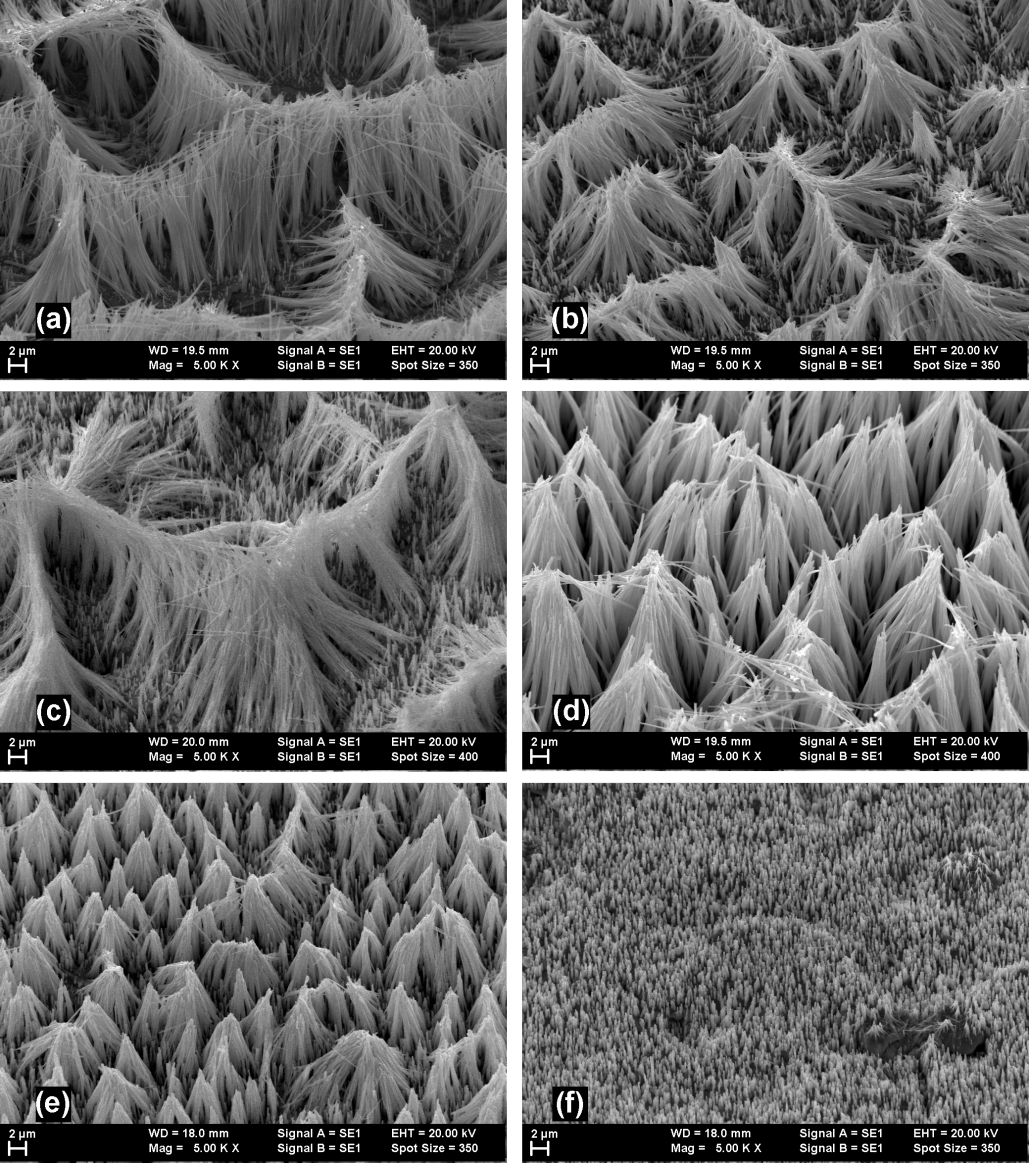
SEM micrographs of CdTe nanowires deposited at (a) −400 mV; (b) −500 mV; (c) −550 mV; (d) −600 mV; (e) −650 mV; (f) −700 mV.

In Figure 1 one can observe SEM images of such arrays of nanowires prepared at different electrode potentials, shown after the host membrane was dissolved. As can be seen from the micrographs, the morphology of the cylindrical nanowires corresponds to the template pore characteristics.

**Figure 2:**
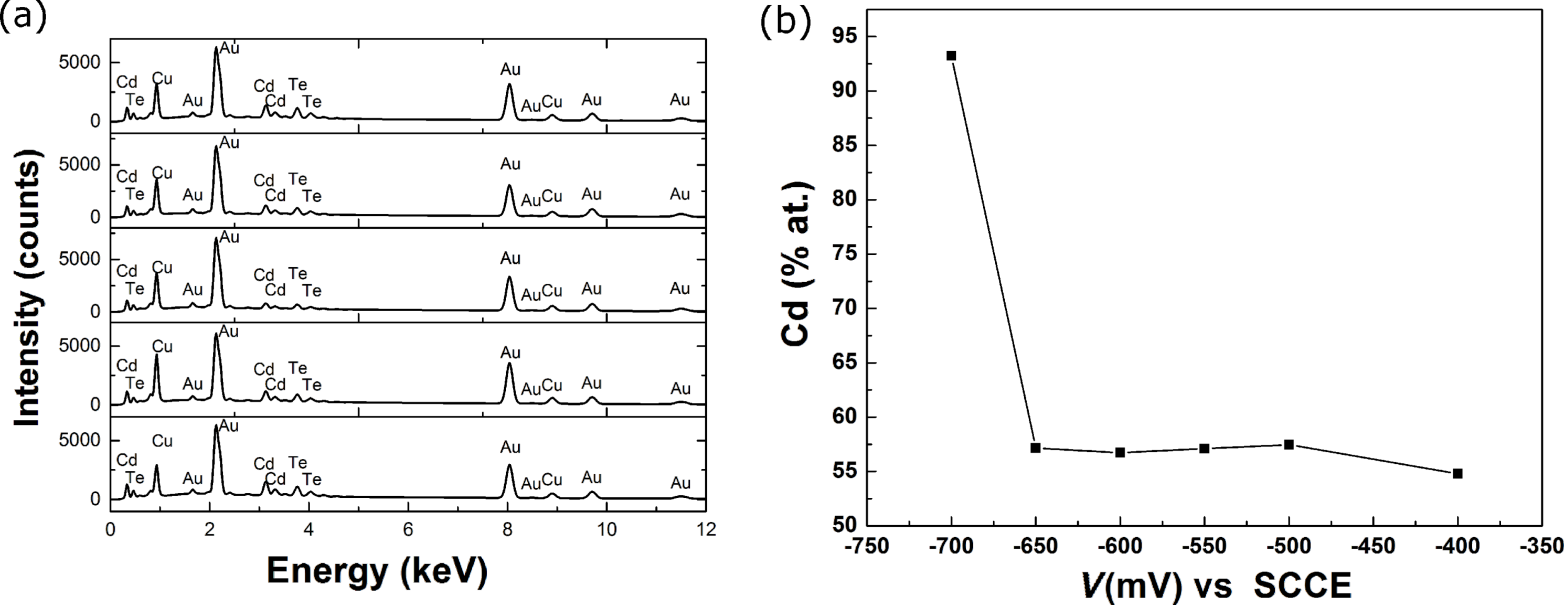
(a) EDX spectra for a series of samples prepared at different electrode potentials; (b) Cd content versus working electrode potential.

EDX spectra deconvolution was employed to determine the composition of the nanowires for different working electrode voltages and the dependence of the composition on the working electrode potential is presented in Figure 2b. A quasi-plateau of the potential for the compound nanowires ranging from −500 to −650 mV vs SCE was found.

**Figure 3:**
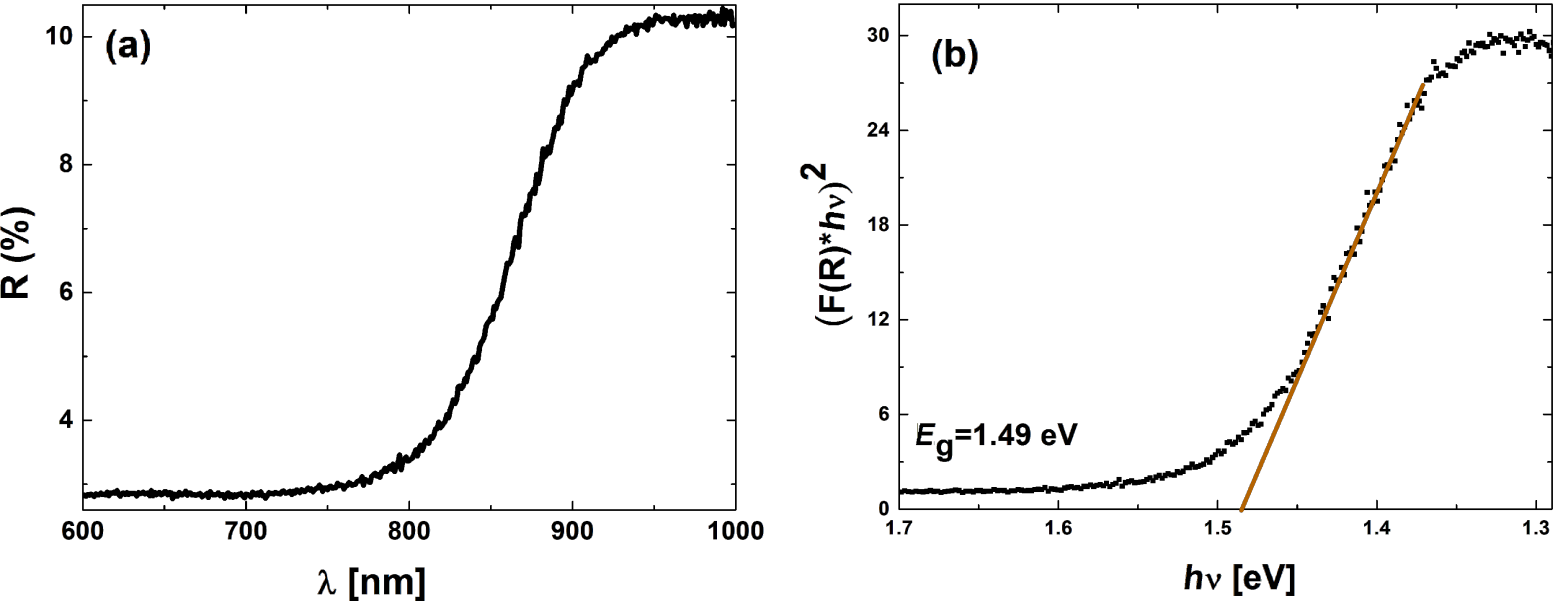
(a) Spectral reflectance curve and (b) Kubelka–Munk representation for band gap determination of CdTe deposited at −500 mV.

The optical properties of compound semiconductors are of high interest since optoelectronic devices are a straightforward application. CdTe is an example of a semiconductor that is extensively employed as an active layer in solar cells and its high absorption coefficient makes it extremely efficient. Figure 3 gives the optical reflection spectra for arrays of CdTe nanowires prepared at different overvoltages. The band gap of the semiconductor was determined to be 1.49 eV by employing the Kubelka–Munk function. This value is in accordance with literature data at approximately 1.45–1.5 eV [18].

**Figure 4:**
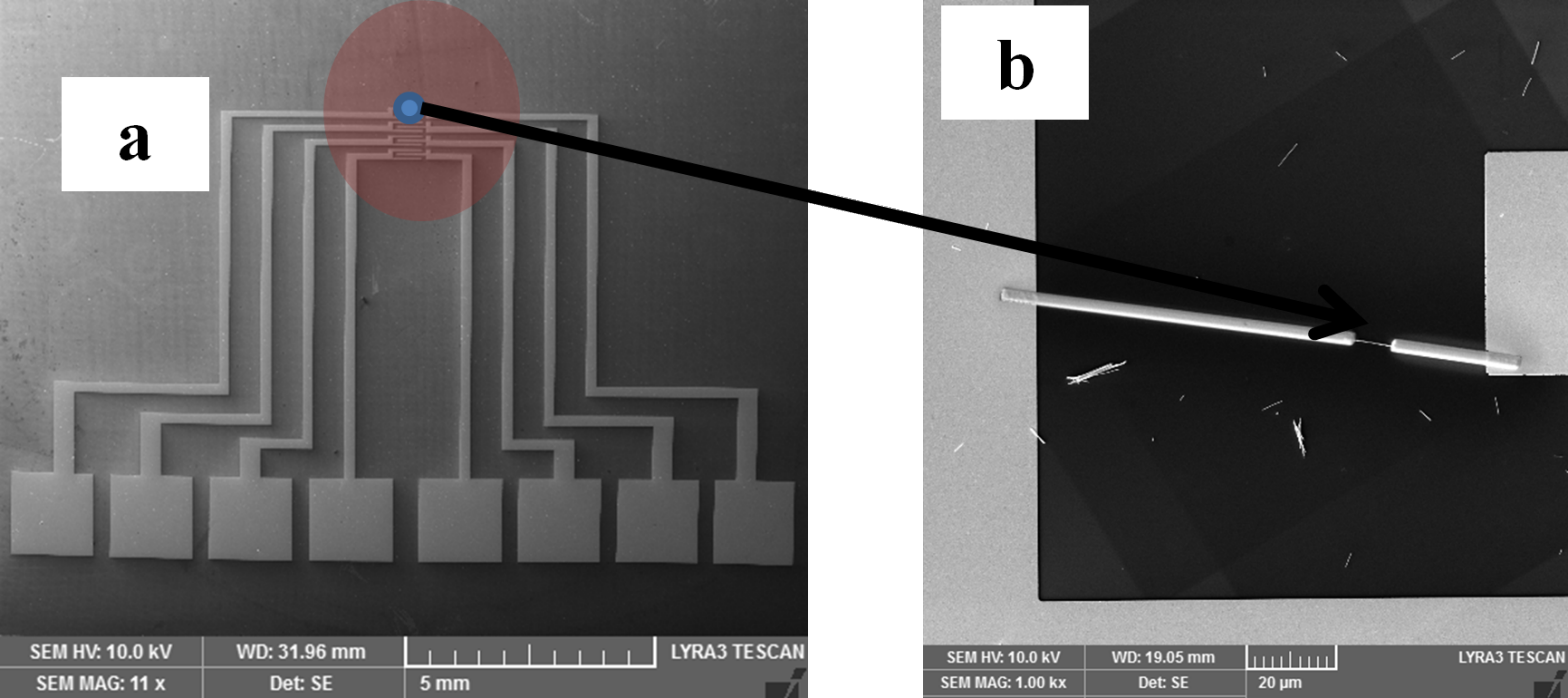
(a) The system of electrodes produced by lithography for contacting the nanowire; (b) an image of an individual nanowire contacted by FIBIM to the larger lithographically prepared electrodes.

The nanowires were further harvested in chloroform by ultrasonication. Taking into account the brittleness of the semiconducting nanostructure, the ultrasonication step was performed for only a few seconds. Further, a droplet of nanowire suspension was placed on Si/SiO_2_ substrates on which interdigitated Ti/Au electrodes were patterned by photolithography (Figure 4).

FIBIM is a direct patterning method employed for the design of metallic nanostructures. The method is based on the interaction of an ion beam with the surface-adsorbed, metal–organic molecules corresponding to the working gas. The decomposition leads to a patterned metallic layer with precisely tailored geometry. In our case, a platinum pattern was formed in order to connect the CdTe nanowires to the photolithographically designed, interdigitated contact (Figure 4b). The drawback of this method is that the metal deposited in this way has a lower conductivity as compared to other deposition methods. This is a direct result of the approach and is due to the presence of carbon in the metal layer. This carbon is a residue due to the organic component of the precursor gas.

Further electrical measurements revealed a slightly nonlinear current–voltage characteristic. When a voltage was applied to the silicon substrate, this gate potential influenced the current through the nanowire.

**Figure 5:**
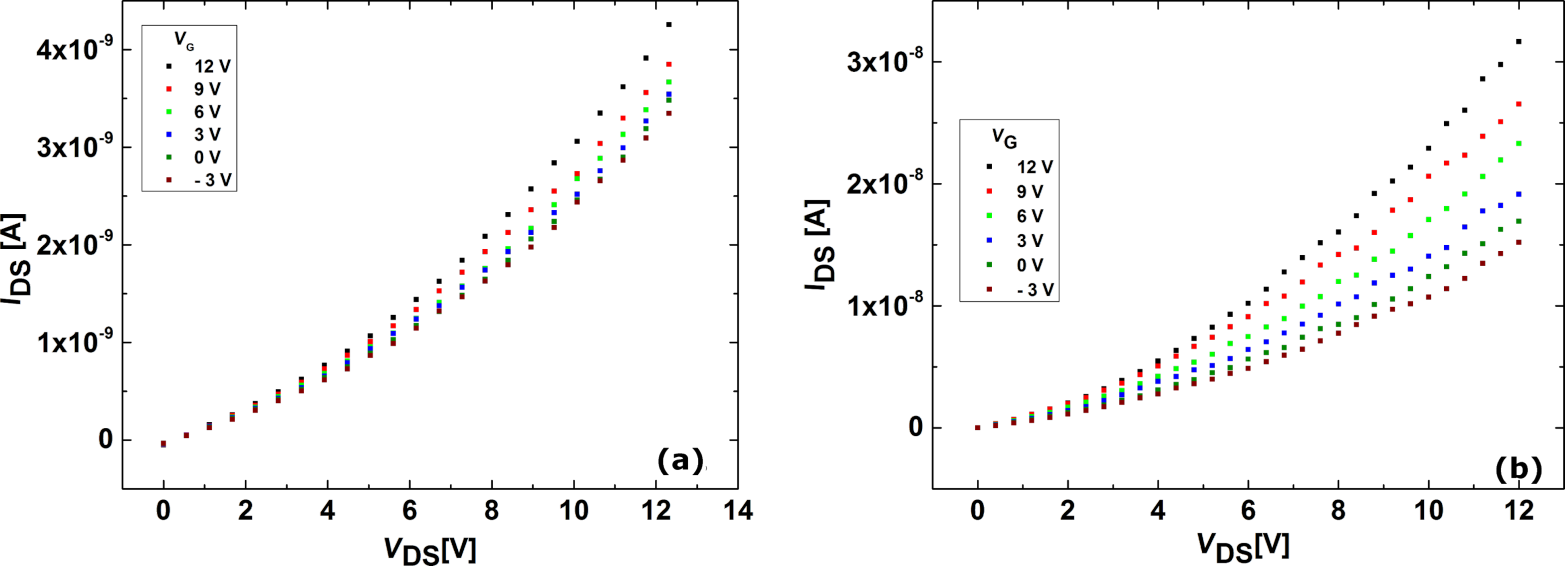
(a) Current–voltage characteristics for a CdTe nanowire contacted by FIBIM; (b) Current–voltage characteristics for a CdTe nanowire contacted by FIBIM after PMMA passivation.

For ZnO it was previously reported [17,19] that a thin layer of polymer covering the nanowire drastically improved the transport properties. This was tested in this work and to our knowledge, only one previous paper dealt with the electrical transport of individual nanowires [20]. No observation of passivation effects on CdTe nanowires was previously reported. We also observed a similar behavior for CdTe, as would be expected for objects with similar geometries. In Figure 5 current–voltage characteristics are presented for a nanowire contacted by this approach before and after poly(methyl methacrylate) (PMMA) passivation.

A difference of almost an order of magnitude between the sample before and after PMMA covering was observed. This increase in current observed when adding the polymer layer has a potential source: the passivation of surface states, which may be responsible for a decrease in the number of the free charge carriers available in the nanowire.

## Conclusion

CdTe nanowires were prepared by electrochemical deposition inside ion track, nanoporous membranes. Substrates with interdigitated electrodes were fabricated by employing a photolithographic approach. After the growth of the nanowires, they were placed onto the substrate and FIBIM was employed to make contacts between the nanowire and the electrodes. The contacts were not pure platinum, but rather a mixture of platinum and carbon, which was the residual phase from the organic component of the gaseous precursor used.

The electrical characteristics were measured and nonlinear I–V characteristics were observed. A strong increase in current was measured when the nanowire was covered with a thin polymer layer. One possible explanation for this phenomenon is that the surface states, which are extremely important for high surface-to-volume ratios, were passivated through the process.

CdTe is an important semiconductor for optoelectronics and this approach to CdTe nanowire fabrication gives the opportunity to measure electrical properties of individual nanostructures and to better understand them in correlation with the preparation methods.

## Experimental

Polycarbonate foils of 30 μm thickness were irradiated with swift heavy ions from the linear accelerator UNILAC at the Gesellschaft für Schwerionenforschung (GSI). The ions (Au, Pb or U) were accelerated at a specific kinetic energy of 11.4 MeV/nucleon. When passing through the polymer foil, each ion leaves a cylindrical defect track as a consequence of its interaction with the target. Further, these tracks were chemically etched, leading to the formation of cylindrical pores. An aqueous solution of 5 M NaOH and 10 vol % methanol was employed for the etching process at a temperature of 50 °C. The etching rate was 2 μm/h, at three minutes of etching, resulting in cylindrical pores of approximately 100 nm diameter.

On one face of the membrane, a thin (50 nm) gold electrode is deposited by means of DC sputtering. This is further strengthened by the electrodeposition of a thick (10 μm) copper film. This membrane was then inserted into an electrochemical cell, with the uncovered side facing the electrolyte.

A potentiostat (Parstat 2272) was employed for controlling the deposition process in a three-electrode setup. The reference electrode was a commercial, saturated calomel electrode and the counter electrode was a 4 cm^2^ platinum foil. A double-walled glass beaker was employed as an electrochemical cell, and a deposition temperature of 80 °C was maintained by means of an external water circulator.

The deposition bath contained CdSO_4_ and TeO_2_ as the sources of the two ions. The pH was adjusted to 2 using sulfuric acid and sodium hydroxide. Higher pHs led to tellurium oxide precipitation.

After nanowire growth, the membrane was dissolved with chloroform leaving the nanowires exposed. The arrays of nanowires fabricated in this manner were investigated by means of scanning electron microscopy, energy dispersive X-ray analysis and optical spectroscopy.

Further, the wires were harvested by ultrasonication into a suspension in chloroform. A droplet of nanowire suspension in semiconductor-grade chloroform was placed on a n^++^ Si/SiO_2_ substrate with patterned, interdigitated contracts. These were obtained by photolithography and sequential deposition of 20 nm of Ti and 200 nm Au. A dual-beam, FIB/FEG machine was employed to connect the individual nanowire with the existing interdigitated contacts. During this FIBIM process, a metal–organic gas containing platinum was injected through a nozzle close to the surface of the sample and decomposed in a precise pattern determined by the Ga ion beam. The result is a stripe of a mixture of Pt–C–Ga with the desired geometry determined by the ion beam scanning pattern.

A probe station was employed for performing the electrical characterization of individual nanowires. In order to investigate the effect of a gate on the transport through the nanowire, a third electrical contact was made to the n^++^ Si substrate. A comparison of the transport properties of the nanowires with and without a passivated thin layer of PMMA was performed. This polymer passivation layer was deposited onto the wire by means of spin coating.
